# Safety and Efficacy of Sorafenib and Lenvatinib in Patients Who Underwent Surgery or Whole-Brain Radiotherapy for Brain Metastasis of Hepatocellular Carcinoma

**DOI:** 10.3390/jcm11061536

**Published:** 2022-03-11

**Authors:** Pang-Shuo Perng, Yu-Hsuan Lai, Po-Hsuan Lee, Chi-Chen Huang, Hao-Hsiang Hsu, Jung-Shun Lee

**Affiliations:** 1 Section of Neurosurgery, Department of Surgery, National Cheng Kung University Hospital, College of Medicine, National Cheng Kung University, Tainan 704, Taiwan; scottperng@gmail.com (P.-S.P.); ftl053@gmail.com (P.-H.L.); charles042085@hotmail.com (C.-C.H.); samhsu1610@gmail.com (H.-H.H.); 2 Department of Oncology, National Cheng Kung University Hospital, College of Medicine, National Cheng Kung University, Tainan 704, Taiwan; coscoscos.tw@hotmail.com; 3 Institute of Clinical Medicine, College of Medicine, National Cheng Kung University, Tainan 704, Taiwan; 4 Department of Cell Biology and Anatomy, College of Medicine, National Cheng Kung University, Tainan 704, Taiwan; 5 Institute of Basic Medical Sciences, College of Medicine, National Cheng Kung University, Tainan 704, Taiwan

**Keywords:** brain metastasis, hepatocellular carcinoma, lenvatinib, sorafenib, surgery, survival, whole-brain radiotherapy

## Abstract

Surgery or whole-brain radiotherapy (WBRT) for the management of brain metastasis of hepatocellular carcinoma (HCC) is associated with improved survival. However, the efficacy of multi-tyrosine kinase inhibitors (TKIs) and possible bleeding complications have not been studied in these patients. Therefore, this study aimed at investigating TKI safety and efficacy in these patients. We retrospectively reviewed 39 patients who underwent surgery or WBRT for brain metastasis of HCC. Intracranial tumor bleeding rates were compared between patients who did and did not receive TKIs. Survival outcomes were analyzed using the log-rank and Cox regression tests. A total of 22 and 7 patients received sorafenib and lenvatinib, respectively. The intracranial tumor bleeding rates were 61.5% and 70% in patients who did and did not receive TKIs, respectively (*p* > 0.99). Survival analysis revealed craniotomy (adjusted odds ratio [AOR]: 0.45, *p* = 0.04), a higher Karnofsky Performance Score (AOR: 0.97, *p* < 0.01), and TKI use (AOR: 0.26, *p* < 0.01) were positive prognostic factors for overall survival. TKIs were associated with better survival outcomes in patients who underwent surgery or WBRT for brain metastasis of HCC and did not increase intracranial bleeding. Therefore, TKIs are efficacious and safe for treating brain metastasis of HCC.

## 1. Introduction

Hepatocellular carcinoma (HCC) is the most common primary tumor of the liver, and more than 1 million new cases are expected to be diagnosed in 2025 [1]. It is more prevalent in East Asian countries due to endemic hepatitis B virus and hepatitis C virus infections [2] and is the second most common cause of cancer-related deaths in Taiwan [3]. The treatment modalities for early- and intermediate-stage HCC include surgical resection, ablation, and transplantation, according to the Barcelona Clinic Liver Cancer staging system [1]. Advanced HCCs that are ineligible for curative resection or local treatment are conventionally candidates for systemic chemotherapy and have notoriously low response rates (0–18%) [4] and a median survival period of 6.4 months [5].

In the last decade, owing to the development of systemic targeted therapy and immunotherapy, multiple trials have shown promising results in patients with advanced HCC [6]. Sorafenib, a multi-tyrosine kinase inhibitor (TKI), is the mainstay treatment for advanced HCC, and it can prolong the median survival to 10–14.7 months in these patients [7,8]. In a recent phase 3 non-inferiority trial in patients with advanced HCC, lenvatinib exhibited good efficacy compared to that of sorafenib, affording a median survival period of 13.6 months. However, many patients were excluded from the initial trials because of the occurrence of brain metastases [9].

With the prolongation of survival in patients with HCC, central nervous system metastasis in these patients is gaining more attention and its incidence has increased from 0.2% to 7%. [10]. Brain metastasis is suggestive of late-stage events in HCC, with the median survival after the diagnosis of brain metastasis being 4–12 weeks [10]. Given this short median survival period, metastasectomy or whole-brain radiotherapy (WBRT) can be applied in select patients; both of these approaches have been associated with better survival outcomes than those afforded by conservative treatment [11,12]. The previously established prognostic factors for survival include the Karnofsky Performance Score (KPS), Child-Pugh score, and treatment modalities for intracranial lesions [13,14]. Despite the clinical use of TKIs in the last 10 years, the prognostic role of TKIs in patients with brain metastasis of HCC has not been discussed. Moreover, some studies have raised a specific concern about intracranial hemorrhage in TKI-treated patients with brain metastasis and even considered the drugs as contraindicated for the management of HCC with brain metastasis [15,16]. Hence, in this study, we aimed to explore the safety and efficacy of TKIs for patients with HCC brain metastasis who had undergone craniotomy for tumor excision or WBRT, in terms of overall survival.

## 2. Methods

### 2.1. Patient Selection

**Figure 1:**
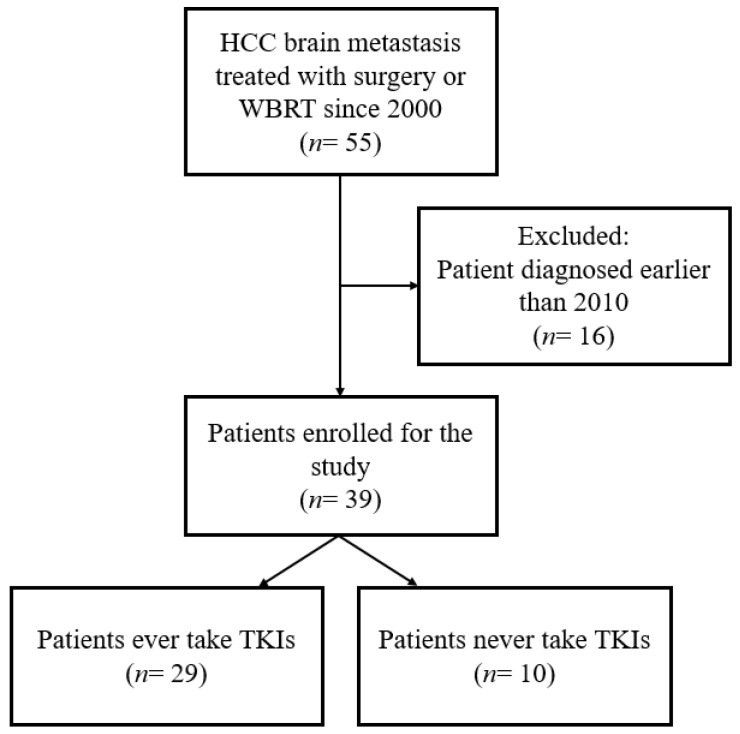
The flow diagram for patient enrollment. HCC: hepatocellular carcinoma; WBRT: whole-brain radiotherapy; TKI: sorafenib and lenvatinib.

Patients who were treated surgically with adjuvant radiotherapy or WBRT for brain metastasis of HCC between January 2010 and August 2021 were included in this study (Figure 1). The patients were treated with sorafenib or lenvatinib as first-line therapy when diagnosed with advanced HCC or brain metastasis. Only patients who received sorafenib or lenvatinib for at least 4 weeks with a full follow-up were considered as having received TKI treatment [17]. Sorafenib has been approved in our national health insurance system since 2010; therefore, in this study, data were only recorded from 2010 onward. Patients who could not tolerate the side effects even after dosage adjustment, participated in clinical trials for other first-line therapies such as nivolumab or chemotherapy, and refused treatment were considered as not treated with TKIs. The indications of surgery for brain metastasis were symptomatic and surgically accessible lesions included single lesion or multiple lesions with a large tumor (>3 cm) producing mass effect [18]. Adjuvant radiotherapy was followed by craniotomy for better local tumor control. WBRT was administered if surgery was not indicated or if the patient opted for it.

### 2.2. Follow-Up Outcomes and Data Collected

Brain computed tomography was conducted once patients had change of consciousness in order to rule out intracranial hemorrhage and screen for the possibility of central nervous system lesions. Gadolinium enhanced magnetic resonance imaging (MRI) was performed in every patient once brain metastasis was suspected. Intra-tumoral hemorrhage was detected by hypointensity on susceptibility-weighted imaging and gradient echo sequences and confirmed by pathological diagnosis if surgery was indicated. Coagulation function and platelet count were evaluated at an interval of 3 months and were treated if the patient had a bleeding tendency or experienced bleeding events such as variceal bleeding.

The clinical data used for calculation were reviewed from the time of diagnosis of brain metastasis. The variables reviewed included age, sex, alcohol consumption, extracranial metastasis state, Child-Pugh score, KPS, brain metastasis-related tumor number, tumor size, use of surgical or non-surgical treatment, and use of TKIs or chemotherapy. Overall survival was defined as the period from the date of surgery or the date of WBRT completion to the patient’s death or last follow-up. The study was approved by the institutional review board of our institute. Individual patient consent was not required and therefore was not obtained.

### 2.3. Statistical Analysis

Basic demographic data are presented as mean and standard deviation values or median values with interquartile ranges (IQRs). Log-rank regression and Cox regression methods were used to obtain the Kaplan–Meier survival curve and create a model to predict survival outcomes. Univariate analysis was applied to select the variables for multivariate analysis, with a cutoff *p*-value of 0.2. The results were 2-sided, and statistical significance was set at *p* < 0.05. SPSS 22 software was used for statistical analysis.

## 3. Results

### 3.1. General Demographic Characteristics

In all, 39 patients were included in the present study, and their basic information is listed in Table 1. A total of 22 (56%) patients received sorafenib, and 7 (18%) received lenvatinib. The median Child-Pugh score in the current study was 6 (IQR 5–7). Moreover, 29 (78%) patients had received treatment at the primary site, with 21 (54%) receiving transarterial chemoembolization or hepatic arterial infusion chemotherapy and 24 (62%) undergoing liver resection. Only 3 (8%) patients were diagnosed with HCC and brain metastasis at the same time; in contrast, 36 (92%) patients had metachronous brain metastasis. At the time of brain metastasis diagnosis, 34 (87%) patients had extracranial metastases. As for brain metastasis, the median tumor number was 2 (1–3), with median size being 3.2 ± 1.5 cm; most tumors (87%) were located in the supratentorial area.

### 3.2. Safety of the TKIs

The patients were classified into the following three groups. The first group of patients started taking the TKIs when the brain metastasis was diagnosed, the second group started receiving the TKIs after surgery or WBRT, and the third group of patients had already been using TKIs but were considered to have discontinued them when the brain metastasis was diagnosed. The corresponding intratumoral bleeding rates were 61.5%, 66.7%, and 66.7%, with the intergroup differences between them insignificant (Table 2). In contrast, the bleeding rate was 66.7% in patients who did not receive TKIs. There was no statistical difference in intra-tumoral bleeding between patients taking TKIs and those who did not. Only 1 patient in the cohort (3.6%) underwent surgery for lesion excision and hemostasis for massive intracranial bleeding.

### 3.3. Survival and Prognostic Factor Analysis

**Figure 2:**
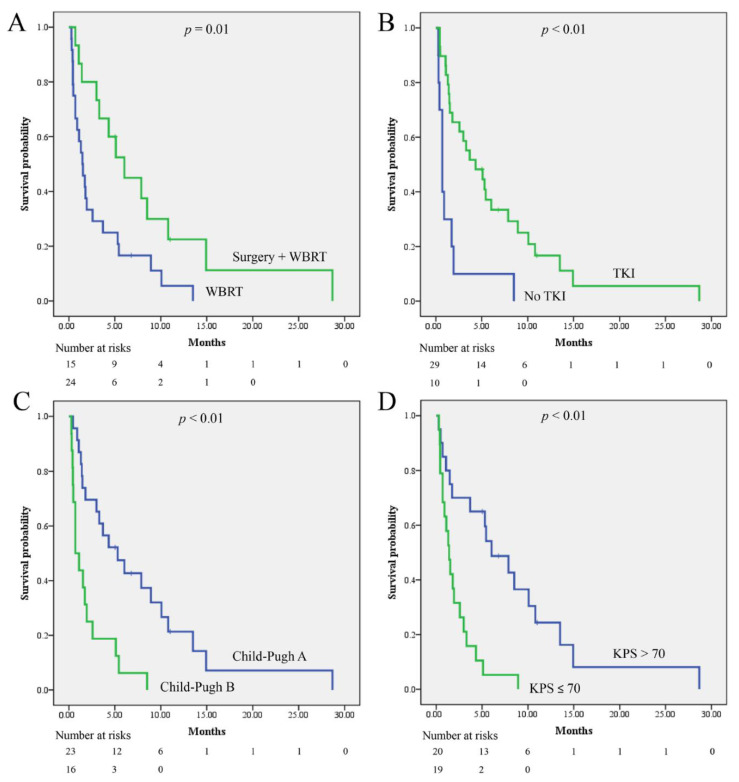
The Kaplan–Meier curves for survival probability were calculated for patients (**A**) having brain metastasis treated with surgery + WBRT or WBRT; (**B**) receiving sorafenib and lenvatinib or not; (**C**) with different Child-Pugh score; (**D**) with different KPS. WBRT: whole-brain radiotherapy; TKI: sorafenib and lenvatinib; KPS: Karnofsky Performance Score.

The median survival for the entire cohort was 3 (1.0–7.0) months. The median survival period for patients having brain metastasis treated with surgery and WBRT were 6.0 (95% CI: 3.1–9.0) and 1.5 (0.7–2.2) months, respectively (*p* = 0.01). In patients who did and did not take TKIs, the median survival periods were 4.3 (1.2–7.4) and 0.7 (0.4–1.0) months, respectively (*p* < 0.01). The median survival periods for the patients with Child-Pugh scores corresponding to classes A and B were 5.3 (1.8–8.7) and 0.7 (0.1–1.3) months, respectively (*p* < 0.01). The median survival periods of patients with KPS > 70 and KPS ≤ 70 were 6.0 (2.6–9.4) and 1.4 (0.8–2.0) months, respectively (*p* < 0.01). The Kaplan–Meier curves are presented in Figure 2. Univariate analysis using the Cox regression method showed that higher KPS, use of TKI therapy, and surgery for the management of brain metastasis were associated with improved survival outcomes, while higher Child-Pugh scores were not (Table 3). Multivariate analysis revealed that higher KPS, TKI therapy, and surgery for the management of brain metastasis were independent factors associated with improved overall survival (Table 4).

## 4. Discussion

In this retrospective study, we reviewed the safety and efficacy of TKIs among patients who underwent craniotomy or WBRT for brain metastasis of HCC. The median survival period of the entire cohort was 3 (1.0–7.0) months, which was similar to that reported in most series of HCC brain metastasis [10]. TKIs, including sorafenib and lenvatinib, along with favorable prognostic factors including higher KPS and surgical treatment have been found to be associated with better survival outcomes in such patients. In addition, the tumor bleeding rates were similar between the patients who received TKIs and those who did not. To our knowledge, this is the first study to discuss the rate of intracranial tumor hemorrhage and prognostic role of sorafenib and lenvatinib in patients with brain metastasis of HCC.

Sorafenib was previously used in the management of advanced renal cell carcinoma and was proven to prolong the progression-free survival from 2.8 to 5.5 months compared to placebo [19]. Owing to its anti-proliferative and anti-angiogenic effects, sorafenib has also been studied in the case of primary central nervous system tumors. Sorafenib has been shown to significantly suppress the growth of intracranial gliomas and increase the sensitivity of glioma cells to temozolomide in vitro [20,21]. On the other hand, the new-generation TKI, lenvatinib, has more potent activity against vascular endothelial growth factor and fibroblast growth factor receptors. In vivo studies on brain metastasis of thyroid cancers and advanced glioblastomas demonstrated that lenvatinib significantly inhibited tumor growth [22,23]. Taken together, the findings suggest that TKIs can be effective in the management of different grades of gliomas and specific brain metastases. Theoretically, since the molecular sizes of sorafenib and lenvatinib are 465 and 426 Da, respectively; both agents can pass through the blood–brain barrier. The brain penetration of sorafenib was reported to be 3.4–9.4% in studies conducted in monkeys and rats [24,25]. Moreover, Wang et al. showed that lenvatinib exhibited better penetration of the blood–brain barrier than did sorafenib [10]. We propose that the intrinsic central nervous system penetration and antitumor effect of TKIs are the main factors that contribute to the better survival outcomes in our study.

Clinically, one of the major concerns associated with the use of TKIs is an increased hemorrhage rate, such as in the case of liver tumor bleeding in patients with HCC, and intracerebral hemorrhage in patients with brain metastasis of renal carcinoma [16]. The mechanism for intratumoral bleeding was inferred to be the weakening of the vascular wall due to reduced endothelial cell regenerative capacity [26]. Another hypothesis was the disturbance of platelet–endothelial interaction and homeostasis by TKIs [27]. The bleeding risk of patients receiving sorafenib and lenvatinib is currently under debate. A retrospective study of 252 patients with HCC in Japan showed no increased bleeding risk in patients taking sorafenib [28]. A meta-analysis conducted by Dai et al. revealed an increased risk of low-grade hemorrhagic events but not of high-grade hemorrhagic events in 4720 cancer patients treated with sorafenib [29]. Regarding lenvatinib, although some case reports and cohort studies have shown increased intratumoral bleeding in patients having HCC treated with lenvatinib [30,31], a recent network meta-analysis showed neither lenvatinib nor sorafenib is associated with an increased bleeding incidence in cancer patients [32]. Intratumoral bleeding in the brain can be lethal and requires special attention due to severe mass effects or uncontrolled intracranial hypertension that is different from primary tumor bleeding. Brain metastasis of HCC is known to be prone to intracranial tumor bleeding, with an incidence ranging from 39.5 to 66.7% [12,33]. Therefore, any medications associated with superimposed intracranial hemorrhage can result in disastrous consequences in patients with brain metastasis of HCC. Our results showed that the bleeding rates in patients who never received TKIs and those who were taking TKIs were 70% and 61.5%, respectively (*p* > 0.99). Therefore, we consider the use of multi-targeted TKIs in patients with brain metastasis of HCC a safe strategy.

Surgical resection of brain metastases was associated with better survival outcomes when compared to those with WBRT alone in our study (Table 4). Surgery has been the mainstay treatment for brain metastasis. A recent study in the United States showed that resection of brain metastasis combined with radiosurgery was associated with increased survival compared to that afforded by radiosurgery alone (10.9 vs. 2.8 months; *p* = 0.04) [34]. Park et al. reviewed 59 patients with brain metastasis of HCC in Korea and found that surgical resection afforded better median survival than that afforded by WBRT and gamma knife surgery, (14.7 vs. 4.3 vs. 5.3 weeks) [14]. Lesionectomy can directly decrease the tumor burden and the mass effect of the brain tumor immediately and allow for pathological testing compared to WBRT alone. However, advanced liver disease may be associated with coagulopathy and thrombocytopenia, which are contraindications for cranial surgery. Therefore, the existence of allocation bias cannot be ignored because the surgery group is in a better general condition than is the radiation group. Thus, HCC brain metastasis is not a contraindication for craniotomy; instead, with careful patient selection, surgery can improve functional and survival outcomes.

In addition to surgery, postoperative adjuvant radiotherapy is mandatory for brain metastasis from various tumor origins. Conventionally, adjuvant WBRT could mitigate tumor recurrence at the surgical sites. However, the WBRT-related mental decline is a growing issue, as the overall survival in those patients is longer than that previously [35]. To address this issue, several radiation modalities have been developed. In a recent randomized controlled trial on the application of stereotactic radiosurgery (SRS) for patients with brain metastasis, regardless of the original cell types, the results showed that SRS exerts the same beneficial effects on overall survival with a lesser decline in cognitive function as compared to those with WBRT [36]. Another newly developed technique is hippocampal avoidance WBRT and memantine, which better preserves cognitive function in brain metastatic patients with no metastases to the hippocampal region [37]. The local recurrence rate for brain metastasis was decreased to 10–12.5% after utilizing adjuvant radiation therapy [38]. In contrast to brain metastasis from lung or breast cancers, recurrent brain metastasis from HCC after craniotomy and adjuvant WBRT is usually not an issue owing to the short survival after the first brain metastasis. However, if necessary, re-operation or salvage radiation therapies could still be applied for selected patients in a similar manner as that used for other brain lesions [39].

Previous researchers have substantiated the idea that patients with better underlying conditions, including higher KPS and low Child-Pugh scores, are associated with better survival outcomes [11,13]. The results of our study were similar. The selection criteria for aggressive treatment of HCC brain metastasis are not well-established, owing to the rarity of brain metastasis in patients with HCC. Mostly, we treated those patients by consensus, as in cases of other types of brain metastasis. However, end-stage liver disease often presents with refractory ascites, cachexia, a poor appetite, and encephalopathy, all of which lead to poor quality of life. It is mandatory to weigh the potential benefits of aggressive treatment and the possible complications and the remaining life span to make an individualized treatment plans for each patient. Comprehensive discussions involving neurosurgeons, hepatologists, oncologists, radiation oncologists, and patients are necessary before proceeding with any treatment.

This study has several limitations. First, although this was a pilot study, it was a single-center study with a limited number of patients. Second, although TKIs can theoretically pass the blood–brain barrier, the exact rate of cerebral spinal fluid penetration for TKI therapy was unavailable in our study. Third, stereotactic radiosurgery was not yet a treatment modality for HCC brain metastasis at our institute; therefore, the data were lacking. Fourth, the actionable mutations for HCC and different inherited tumor behavior were not accessed in our study, and this might have led to some bias during the analysis.

## 5. Conclusions

TKIs, including sorafenib and lenvatinib, were associated with better survival outcomes in patients who underwent surgery or WBRT for brain metastasis of HCC. In addition, the intracranial tumor bleeding rate did not increase in patients who took TKIs. Thus, TKIs are effective and safe for the treatment of brain metastases of HCC.

## Figures and Tables

**Table 1:** General demographic.

		*n*
Age, median (IQR)		59 (49–63)
Sex, *n* (%)	Male	34 (87)
	Female	5 (13)
Child-Pugh classification, *n* (%)	A	23 (59)
	B	16 (41)
KPS, *n* (%)	>80	8 (21)
	70–80	19 (49)
	<70	12 (30)
Alcohol history, *n* (%)		11 (28)
Hepatitis, *n* (%)	hepatitis B virus	29 (74)
	hepatitis C virus	10 (26)
	Non B-Non C	5 (13)
Extracranial metastasis, *n* (%)		34 (87)
Brain metastasis number, *n* (%)	1	19 (49)
	2	9 (23)
	>3	11 (28)
Brain metastasis size (cm), mean ± SD		3.2 ± 1.5
Brain metastasis location	Supratentorium	34 (87)
	Infratentorium	5 (13)
Timing to brain metastasis, *n* (%)	Synchronous	3 (8)
	Metachronous	36 (92)
TKI therapy, *n* (%)	Sorafenib	22 (56)
	Lenvatinib	7 (18)
	None antiangiogenic	10 (26)
Brain metastasis treatment, *n* (%)	Surgery + WBRT	15 (38)
	WBRT	24 (62)
Localregional therapy, *n* (%)	TACE or HAIC	21 (54)
	Liver resection	24 (62)

KPS: Karnofsky Performance Score; WBRT: Whole-brain radiotherapy; TACE: Transarterial chemoembolization; HAIC: Hepatic arterial infusion chemotherapy; IQR: Interquartile range; SD: Standard deviation. TKI: multi-tyrosine kinase inhibitor.

**Table 2:** Intra-tumoral bleeding rate in brain metastasis.

		*n*	Tumor Bleeding	%	*p* Value
Patients never received TKI		10	7	70	Reference
Patients received TKI	Continuously	13	8	61.5	>0.99
	Started after surgery or WBRT	6	4	66.7	>0.99
	Withdrawal	10	7	70	>0.99

TKI: multi-tyrosine kinase inhibitor, including sorafenib and lenvatinib; WBRT: whole-brain radiotherapy.

**Table 3:** Univariate analysis for overall survival.

		*n*	Hazard Ratio	95% CI		*p* Value
				Lower	Upper	
Age		39	0.99	0.96	1.03	0.77
Sex	Male	34				
	Female	5	0.98	0.37	2.56	0.96
Child-Pugh calssification	A	23				
	B	16	3.56	1.7	7.44	0.001
Karnofsky Performance Score		39	0.96	0.94	0.99	0.003
Alcohol history		11	1.35	0.65	2.81	0.42
Hepatitis B infection	Not carrier	10				
	Carrier	29	1.01	0.47	2.18	0.98
Hepatitis C infection	Not carrier	29				
	Carrier	10	1.25	0.58	2.7	0.58
Extracranial metastasis		34	1.86	0.63	5.53	0.26
Brain metastasis number	1	19				
	2	9	1.67	0.7	4	0.25
	>3	11	2.24	0.98	5.1	0.06
Brain metastasis size		39	0.93	0.75	1.15	0.48
Timing to brain metastasis	Synchronous	3				
	Metachronous	34	0.95	0.29	3.13	0.93
Brain metastasis location	Supratentorium	34				
	Infratentorium	5	3.3	1.2	9.2	0.023
TKI therapy	Sorafenib or Lenvatinib	29	0.28	0.13	0.62	0.002
Metastasis treatment	WBRT	25				
	Surgery + WBRT	12	0.4	0.19	0.83	0.014
Localregional therapy		31	0.61	0.27	1.36	0.23

CI: Confidence interval; WBRT: Whole-brain radiotherapy; TKI: multi-tyrosine kinase inhibitor, including sorafenib and lenvatinib.

**Table 4:** Multivariate analysis for overall survival.

		*n*	Adjust Odds Ratio	95% CI		*p* Value
				Lower	Upper	
TKI therapy	Sorafenib or Lenvatinib	29	0.26	0.12	0.6	0.001
Metastasis treatment	WBRT	25				
	Surgery + WBRT	12	0.45	0.21	0.97	0.041
Karnofsky Performance Score		39	0.97	0.94	0.99	0.006

CI: Confidence interval; WBRT: Whole-brain radiotherapy; TKI: multi-tyrosine kinase inhibitor, including sorafenib and lenvatinib.

## Data Availability

The data presented in this study are available on request from the corresponding author. The data are not publicly available due to privacy.
